# Comprehending the impact of low vision on the lives of children and adolescents: a qualitative approach

**DOI:** 10.1007/s11136-016-1292-8

**Published:** 2016-04-13

**Authors:** Linda Rainey, Ellen Bernadette Maria Elsman, Ruth Marie Antoinette van Nispen, Lisette Michelle van Leeuwen, Gerardus Hermanus Maria Bartholomeus van Rens

**Affiliations:** 1 Department for Health Evidence, Radboud University Medical Centre, Nijmegen, The Netherlands; 2 Department of Ophthalmology, VU University Medical Centre, EMGO+ Institute for Health and Care Research, PO Box 7057, 1007 MB Amsterdam, The Netherlands; 3 Section Audiology, Department of Otolaryngology-Head and Neck Surgery, VU University Medical Centre, Amsterdam, The Netherlands; 4 Department of Ophthalmology, Elkerliek Hospital, Wesselmanlaan 25, 5707 HA Helmond, The Netherlands; 5 VU University Medical Centre PK4X187, PO Box 7700, 1000 SN Amsterdam, The Netherlands

**Keywords:** Visual impairment, Children, Adolescents, Concept mapping, International Classification of Functioning, Disability and Health for Children and Youth

## Abstract

**Purpose:**

To develop a comprehensive, conceptual model detailing the aspects of a child’s life (<18 years) that are affected by low vision.

**Methods:**

Three stakeholders were involved in the developmental process of the conceptual model: children and adolescents with a visual impairment (*n* = 40), parents of children with a visual impairment (*n* = 25) and professionals of multidisciplinary rehabilitation centres and specialised schools (*n* = 25). Qualitative methods including focus groups, online and face-to-face brainstorming sessions and concept mapping were used to investigate the impact of visual impairment on the lives of children and adolescents and to create the conceptual model. To aid interpretation of the large age range, four age-bands were formed.

**Results:**

For each age-band (0–2, 3–6, 7–12 and 13–17 years), a total of 153, 200, 297 and 306 statements were generated by all stakeholders, respectively. The conceptual models show that low vision affects the sensorial development as well as the physical, psychological and social well-being of children and adolescents. In addition, identified external factors (i.e. education/employment and parental influence) can either facilitate or hinder participation.

**Conclusions:**

The developed model shows which life aspects of children are affected by low vision. The needs identified by children and adolescents correspond not entirely to the perspective of parents and low vision professionals. Future research should focus on developing and validating a new questionnaire based on the conceptual model. This will aid goal setting, rehabilitation referral and the accomplishment of developmental milestones and life transitions of children and adolescents with a visual impairment, ultimately improving their participation and quality of life.

**Electronic supplementary material:**

The online version of this article (doi:10.1007/s11136-016-1292-8) contains supplementary material, which is available to authorized users.

## Introduction

Low vision is estimated to affect around 2600 children aged 0–14 years in the Netherlands (total population approximately 17 million) [1]. The main causes of low vision among Western children are developmental and genetic disorders [2]. Good visual skills are required in child development when acquiring cognitive and functional skills [3]. In addition, low vision is associated with a delay in motor development and poorer mathematical, social and immediate problem-solving skills [4–7]. Khadka et al. [8] showed that, although children and adolescents with a visual impairment have lifestyles similar to that of their sighted peers, they experience participation restrictions in the areas of leisure time and sports and restrictions with respect to social interaction. Moreover, starting and sustaining social and romantic relationships and interactive play is hindered by low vision in children and adolescents [9–12]. Rehabilitation services can facilitate improvements in functional status, participation in activities and quality of life; however, little is known about participation goals in children and adolescents with low vision [13].

Throughout the developmental process, children experience rapid and significant transitions, e.g. acquiring new skills and knowledge, educational transitions and physiological changes. Therefore, the planning of rehabilitation services for children should be set within a long-term perspective and should provide support to children and their families, discussing the affected life areas and participation restrictions prior to and during rehabilitation [14]. Although the capacity of low vision to impact the lives of children and adolescents, and the importance of rehabilitation is well recognised, a structural and systematic understanding of which participation domains are affected in children and adolescents is lacking. This can negatively affect the first step of rehabilitation: setting participation goals during an intake procedure. Multidisciplinary Rehabilitation Centres (MRCs) are currently dependent on their own knowledge and clinical expertise when creating an inventory of a child’s or adolescent’s participation goals. Relying solely on the personal expertise of low vision professionals (e.g. social workers, occupational therapists, educational and developmental psychologists) carries a risk of bias and an underrepresentation of needs [15].

The International Classification of Functioning, Disability and Health for Children and Youth (ICF-CY) offers a conceptual framework to identify problems in infancy, childhood and adolescence [16]. The ICF-CY aims to identify problems in body structure and function, activity limitations, participation restrictions and environmental factors of importance to children and youth [16]. A patient-record study evaluating participation goals of visually impaired children and adolescents (aged 0–18 years) obtained during the intake procedure, showed that most goals could be linked to the structure of ICF-CY, supporting its suitability as a general framework for assessing life areas affected by low vision in children and adolescents [17]. However, goals related to psychological functioning (e.g. coping with or adapting to the impairment) could not be linked to its content. In addition, several of the important life areas identified by the ICF-CY (e.g. social interaction, communication and leisure activities) were often not represented in the participation goals of visually impaired children and adolescents [17]. Although this could indicate that low vision does not affect these life areas, this is counterintuitive and not supported by the literature. Moreover, incomplete inventories of participation goals can affect referral to low vision treatment programmes and the quality of care provided [18]. In addition to the general framework of the ICF-CY, it is necessary to develop a comprehensive, conceptual model, detailing the aspects of a child’s life and participation restrictions affected by low vision at different stages (0–17 years). This will aid the rehabilitation process and ultimately improve the participation prospects of children and adolescents with a visual disability and their quality of life. The present study details the development of a conceptual model of the life areas affected by low vision in children and adolescents (0–17 years), involving children and adolescents with a visual impairment, parents and low vision professionals. This paper focuses on visually impaired children and adolescents’ participation in activities, and participation needs and restrictions experienced by them. To facilitate interpretation, four age-bands were formed based on WHO criteria: ≤2, 3–6, 7–12 and 13–17 years. The study protocol was approved by the Medical Ethical Committee of the VU University Medical Centre, Amsterdam, the Netherlands. This study has been performed in accordance with the ethical standards as laid down in the Declaration of Helsinki. Informed consent was obtained from all individual participants included in the study.

## Methods

### Participants

Children and adolescents were recruited from three low vision schools and MRCs for the visually impaired between March and July 2014. Criteria for inclusion in the study were: the child/adolescent was registered at an MRC, aged 7–17 years, had adequate cognitive abilities according to MRC’s registers and had adequate understanding and mastery of the Dutch language.

Parents were selected for recruitment in collaboration with MRCs; children of all participating parents were either receiving or had received rehabilitation services in the past. Parents were included in the study if they had at least one child aged 0–17 years with a visual impairment. In addition, parents needed to have adequate cognitive ability and adequate understanding and mastery of the Dutch language.

Low vision professionals were employees from MRCs and a university hospital. All professionals were specialised in working with visually impaired children and adolescents.

### Brainstorm

#### Children and adolescents

To assess the needs and priorities of children and adolescents, focus group discussions and interviews using a semi-structured format were conducted. The semi-structured format was the same for focus group discussions and interviews and included questions about hobbies, activities at home and at school and social relationships. Questions were not limited to specific activities that could be impaired by low vision, but were directed at all activities the child (would) like(s) to participate in, in order to generate a broad overview. Six focus group discussions were organised to facilitate group brainstorm and discussion. In total, 38 children and adolescents, divided by age-band (7–12 and 13–17 years), participated in a focus group discussion. For two adolescents it was not possible to participate in a focus group discussion because of logistical reasons. Therefore, an individual semi-structured interview was conducted with each of these two adolescents. Table 1 provides details of focus group composition and interviewee characteristics. The focus groups with children and adolescents took place at the children’s schools, whereas the semi-structured interview took place at the children’s homes. Each focus group discussion and interview lasted approximately 60 min. Two moderators, one with a background in developmental psychology, were present. One moderator conducted the focus group discussion or interview and one transcribed the data.

**Table 1:** Details on focus group composition and interviewee characteristics

Type	Number of participants	Age range (mean; SD)	Number of males (%)	Number of blind children^a^ (%)	Type of education
Focus group 1	13	10–15 (11.8; 1.5)	6 (46 %)	2 (15 %)	Low vision school
Focus group 2	9	14–17 (15.7; 1.0)	4 (44 %)	2 (22 %)	Low vision school
Focus group 3	6	10–12 (10.5; 0.8)	4 (67 %)	3 (50 %)	Low vision school
Focus group 4	4	13–16 (14.3; 1.3)	3 (75 %)	1 (25 %)	Low vision school
Focus group 5	4	7–12 (9.0; 2.4)	3 (75 %)	0 (0 %)	Low vision school
Focus group 6	2	7–12 (9.5; 3.5)	1 (50 %)	0 (0 %)	Low vision school
Interview 1	1	16	1 (100 %)	0 (0 %)	Regular education
Interview 2	1	16	0 (0 %)	0 (0 %)	Regular education
Total	40	7–17 (12.5; 2.8)	22 (55 %)	8 (20 %)	

*SD* standard deviation

^a^Blind: visual acuity ≤0.05 and/or visual field ≤10°

#### Parents

During the recruitment process, parents expressed a preference for a digital approach, since this approach was considered less time-consuming and less intensive compared to face-to-face meetings. Therefore, online brainstorm sessions were organised in which they could participate at a time and place of their preference. Each participant was sent an email containing general questions about child characteristics and the seeding statement: “Thinking as broadly as you can: generate statements about activities that your child would like to participate in (either independently or with others)”. The seeding statement was not limited to those activities influenced by a visual impairment, to generate a broad overview of statements and activities. Parents returned their answers to the questions via email.

#### Professionals

Low vision professionals participated in four concept-mapping workshops, which took on average 4 h to complete. Concept mapping is a structured method to identify and organise items relating to a particular construct, which comprises both qualitative and quantitative steps [18]. The first phase, the brainstorm session, was performed face-to-face during workshops, guided by two facilitators to structure the process. First, statements were generated through an individual brainstorm with a similar seeding statement as was used for parents: “Thinking as broadly as you can: generate statements about activities that children/adolescents with a visual impairment would like to participate in, either independently or with others”. The seeding statement was modified four times to include the specific age-band. As the seeding statement administered to the parents, the seeding statement for professionals was not limited to those activities impaired by low vision. Next, all statements were shared with the group.

### Clustering

The transcripts of interviews and focus groups with children and adolescents, and the statements generated by parents were independently and systematically analysed and clustered by two researchers independently. A third researcher was consulted to resolve disagreement between the two researchers. The first step in statement clustering was familiarisation with the data, which involved repeatedly reading the transcripts and statements in an active way, identifying meaning and patterns in the data. Next, statements were organised into meaningful groups, using a data-driven strategy. Themes were developed by evaluating overarching topics and relationships and by studying potential interconnectedness of these topics. Created themes were reviewed by re-reading all statements and evaluating their contribution to the identified themes. At this stage, the validity of identified themes was evaluated by studying the data as a whole and considering the individual contributions to identified themes. By performing a detailed study of the content of each theme, all themes were named and defined. In addition, subthemes were developed if this provided a better explanation of the overall narrative. The clustering phase of the concept-mapping workshop for professionals was performed digitally using a concept-mapping software program (Concept Systems Inc.). Participants were sent instructions via email containing personal log-in information. First, professionals were asked to sort and rate the statements. They sorted statements according to theme and assigned a name to each sort. Then, they rated statements on a scale from 0 to 10 with the question “How important is this statement for the development of the child?”. Based on the obtained data, a concept map was computed for each age-band. The software uses two multivariate statistical methods (multidimensional scaling and cluster analysis) to create the maps. The software analyses patterns among the generated statements that were sorted and rated by the participating professionals, resulting in two-dimensional graphical presentations of item clusters, depicting content similarities and item priority. The software named the clusters in each map, based on the names assigned by the participants. Finally, the concept map was interpreted and revised.

### Integration

To integrate information obtained from children and adolescents, parents and professionals, several steps were undertaken. First, the identified clusters obtained from the statements of children, adolescents and parents, were combined. Second, the generated statements of professionals were graphically represented by four concept maps, one for each age-band. The maximum number of clusters in the concept map (i.e. most specific content analyses) and the minimum number of clusters (i.e. most general content analysis) that provided a sensible and relevant representation of the results were assessed, choosing the number that best reflected the content of all stakeholders. Finally, the clusters and concept maps were combined to create a conceptual model of the affected life domains of children and adolescents with a visual impairment. This integration process was informed by combining the items considered most important by the professionals (judged by the rating process), and the clusters and items most prevalent in the output from children, adolescents and parents.

## Results

### Participants

Forty children with a visual impairment participated in the focus group discussions or semi-structured interviews (response rate: 28.6 %). The average age of the children was 12.5 years (SD = 2.8), 55 % were male, and 20 % were blind according to the criteria of the World Health Organization (i.e. visual acuity ≤0.05 and/or visual field ≤10°).

Twenty-five parents participated in the online brainstorming session (response rate: 10.4 %). Main reasons cited for non-response were too busy and not able to contribute valuable information. The average age of participants’ visually impaired child was 8.8 years (SD = 5.0, range 1–17), 63.2 % of the children were male, and 12.5 % of the children were blind. Parents reported a large variety of causes for low vision in their children, such as uveitis, achromatopsia, congenital cataract and albinism.

A total of 25 professionals participated in the face-to-face concept-mapping workshop; their professions were occupational therapist (*n* = 9), developmental psychologist (*n* = 4), behavioural therapist, neuropsychologist and social worker (each *n* = 3), intaker, education coordinator and ophthalmologist (each *n* = 1).

### Brainstorm

Children aged 7–12 years (*n* = 22; 55.0 %) generated 90 statements during the face-to-face verbal conversations, and children aged 13–17 generated 104 statements. Parents generated 88 statements in total, and professionals generated 65, 112, 119, 114 statements in response to the seeding statement for each age-band (0–2, 3–6, 7–12, 13–17 years), respectively.

### Clustering

The generated statements of children, adolescents and parents resulted in the identification of 16 clusters. Table 2 presents the clusters and the age-specific participation within these clusters. Statements related to “mobility” were mentioned by parents of children in all age-bands, as well as by children and adolescents. Parents of children younger than 12 years generated statements related to “sensory perception”, “orientation” and “play”. Statements related to “school”, “sports and hobbies”, “energy”, “social–emotional well-being” and “acceptance” were mentioned by (parents of) children and adolescents older than 7 years. Statements related to “self-care” were only mentioned by children and adolescents, and only adolescents mentioned statements related to “overprotective parents”.

**Table 2:** Clusters identified from the mind maps from children, adolescents and parents and their age-specific participation

Clusters	Age-specific participation					
	Parents				Children	Adolescents
	0–2 years	3–6 years	7–12 years	13–17 years	7–12 years	13–17 years
Sensory perception	X	X	X			
Processing sensory information					X	
Orientation	X	X	X			
Mobility	X	X	X	X	X	X
Activities of daily living	X	X	X	X		
Self-care					X	X
Communication	X		X		X	X
Social interaction		X	X	X	X	X
School			X	X	X	X
Employment						X
Play	X	X	X			
Sports and hobbies			X	X	X	X
Energy			X	X		
Social–emotional well-being			X	X	X	X
Acceptance			X	X	X	X
Overprotective parents						X

**Fig. 1:**
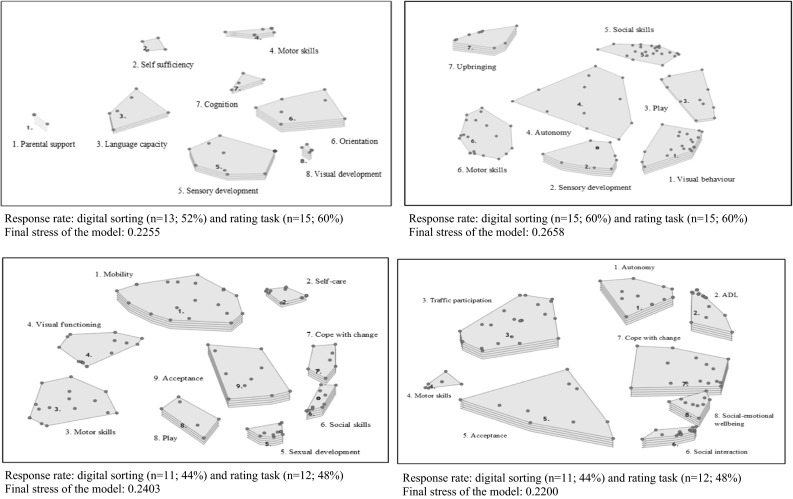
Concept-mapping results of life situations affected by a visual disability, according to low vision professionals; from *left* to *right* age-band 0–2, 3–6, 7–12 and 13–17. The *maps* show clusters of items (the *dots*) that were considered similar in thematic content: more layers indicate greater importance

Figure 1 shows the four concept maps which were formed based on the obtained sorting and rating data of the professionals. The stress of the concept maps ranged from 0.22 to 0.27, indicating good statistical fit to the multidimensional scaling model. The concept maps show that “parental support” and “upbringing” were only mentioned for the first two age categories, reflecting the importance of attachment in those age-bands. “Development of orientation skills” was only mentioned for the youngest children (0–2 years), whereas “visual development” was considered most important in the first 6 years. “Social skills”, “coping skills” and “acceptance of the disability” were rated important for children aged 7 years and older. “Social–emotional well-being” and independently participating in “self-care” and “traffic activities”, as well as “activities of daily living (ADL)”, were considered important for adolescents aged ≥13 years.

 Table 3 in supplementary material presents an overview of all generated statements by children, adolescents, parents and professionals and the (overarching) themes that were identified. Because of overlap, broadness and specificity of the statements generated by children, adolescents and parents, some statements were further divided using the statements generated by professionals. For example, parents generated as a statement that the gross motor development of their child was falling behind. This statement was then further divided using the statements by professionals related to gross motor development, like walking, climbing the stairs and cycling.

### Integration

**Fig. 2:**
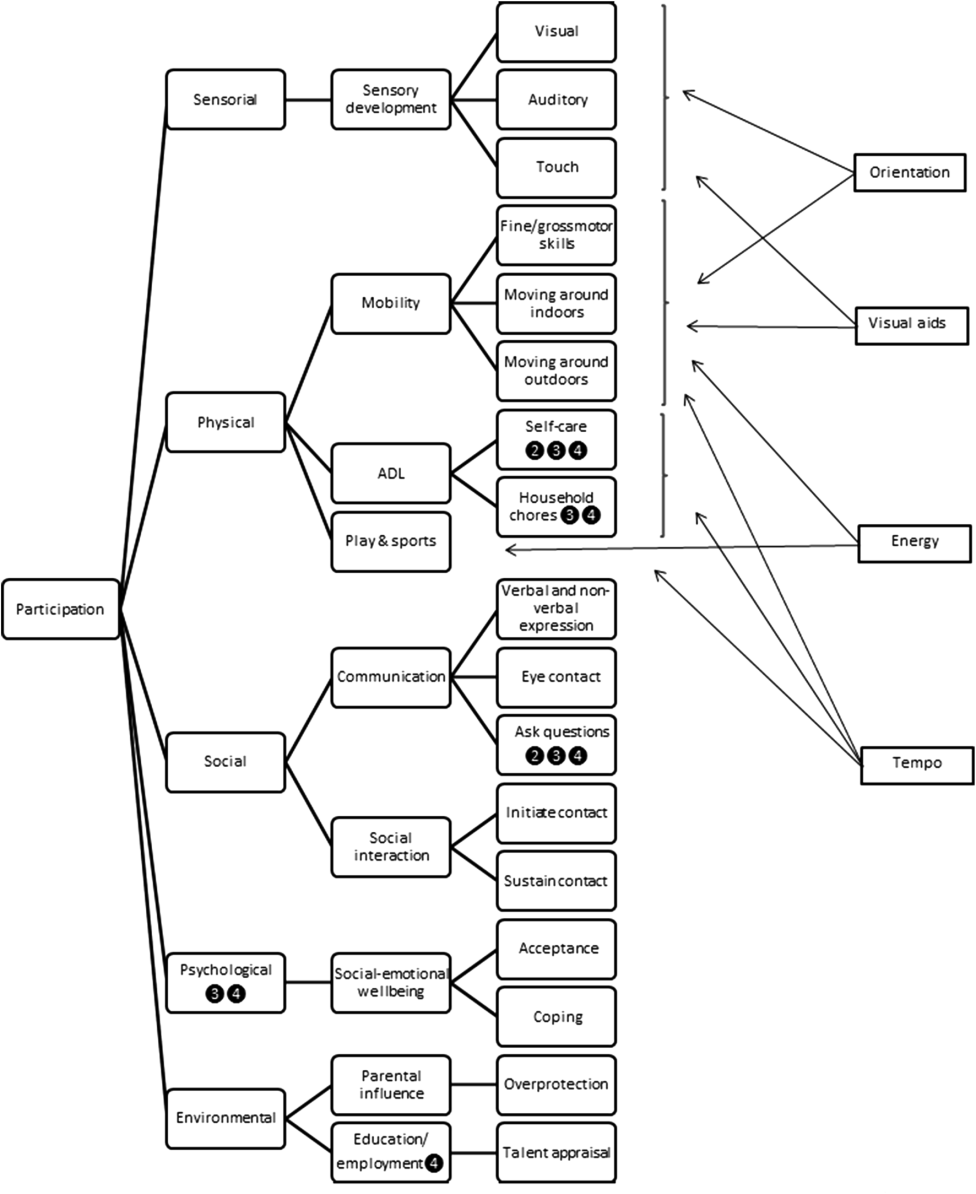
Conceptual model of life areas affected by low vision in children and adolescents. The domains are applicable to all age-bands, with the exception of those indicated with a number (2: 3–6; 3: 7–12; or 4: 13–17)

Figure 2 represents the integration of the clusters and concept maps combined for all stakeholders representing a conceptual model of the life situations affected by low vision in children and adolescents. Table 3 in supplementary material provides insight into how the generated statements contribute to the underlying conceptual model.

Parents were identified by both professionals and children/adolescents as important facilitating or hindering factors in participation. Comparing the input from professionals and parents at item level, it can be discerned that they appraised the affected life domains of children and adolescents similarly; children and adolescents have a different perspective.

#### Age-band 0–2 years

Within this age-band, sensory development is most important, indicated by the high number of statements within this domain. Both parents and professionals described the importance of reaching practical milestones required for healthy development of children aged 0–2 years. However, they also emphasised the impact low vision can have on the psychological well-being of the child. In addition, parents and professionals indicated the complicated attachment process of children.

#### Age-band 3–6 years

For children aged 3–6 years mobile independence and play become more relevant domains where parents subsequently indicate that they struggle to find a balance between encouraging their child’s independence and their instinct to protect their child. Compared to the age-band 0–2 years, more statements were generated concerning parental influence and issues and challenges from parents, for example worrying about their child’s future.

#### Age-band 7–12 years

In this age-band, communication, social interaction and social–emotional well-being become more important, together with school life. According to professionals and parents, the life of children aged 7–12 years is characterised by an increasing awareness of the visual impairment, the importance of appropriate coping techniques and sexual development. Children reported anxiety about asking others for help, feeling very dependent on their parents for ADL, having few friends, feeling lonely and wanting to attend regular education. One child stated: “There are not many children in the neighbourhood, and the few children that live in the neighbourhood do not take into account that I’m visually impaired when they are playing. Therefore, I can’t participate”. Furthermore, children indicated that their social activities often revolve around the family rather than around peers.

#### Age-band 13–17 years

Professionals reported that adolescents have an incessant craving for autonomy and discard any limitations (e.g. with regard to driving a car). In addition, parents indicated that their child has a tendency to overcompensate to be able to keep up with sighted peers, leading to exhaustion. Parents also reported that their child has become socially secluded, i.e. withdrawing from social interaction, because they worry about “not fitting in”, or showing inappropriate behaviour. The social isolation reported by parents was mentioned less frequently by the adolescents themselves, who stressed they find plenty of friends online, i.e. via computer games or social media. Furthermore, they were unfazed by the complexity of household tasks, stating: “We are not stupid, as long as you teach us how to do it, we will be able to”. However, all adolescents expressed great concern about their future life. Although all of them craved autonomy, they were also reluctant. They identified “special education” as an important barrier to full participation, stating that it offers limited opportunities to increase one’s independence. Adolescents were very aware of the “safety” that special education provided them. They stated that the idea of leaving this safe environment sometimes overwhelms them. In addition, they worry about reaching their full potential, the biased appraisal of future employers, living alone in a new environment and being solely responsible for the household (including finances).

## Discussion

The present study aimed to develop a comprehensive framework of the impact of low vision on the lives of children and adolescents. It was found that low vision affects sensorial development and physical, psychological and social well-being of children and adolescents. In addition, external factors that were identified (i.e. education/employment and parental influence) either facilitate or hinder participation. Moreover, this study demonstrated that the views of professionals and parents not always correspond to the perspective of visually impaired children and adolescents. Relying solely on reports of parent and professionals can therefore lead to an underestimation of the assistance required or misplaced emphasis on particular domains during treatment. Even if the right domains are identified, the content of treatment may not necessarily correspond to the real needs of the child/adolescent. Inclusion of the perspective of the child itself is therefore of utmost importance.

The present study found that the first few years (0–2 years) of children appear to be most crucially influenced by a complicated attachment process. Although secure attachment is vital for healthy development, professionals indicated that parents can lack the appropriate skills to establish this secure bond with their child, because this process usually occurs naturally based on visual cues. Since these visual cues can be more difficult (or impossible) for the child to process, the child’s needs have to be compensated using other sensorial processes [19]. For the age-band 3–6 years, mobility and mobile independence are often mentioned. On the one hand, parents want to encourage their child to become independent, but on the other hand they want to protect their child. This is in accordance with studies stating that parents of a visually impaired child tend to discourage him/her from participating in childhood play or sports for fear of injuries [4, 20]. However, professionals emphasised the importance of stimulating the child to enable him/her to become familiar with objects and situations that could otherwise instil anxiety. For the age-band 7–12 years, both professionals and parents mentioned general development and increased awareness of the visual impairment. Additionally, professionals mentioned sexual development, while children mentioned other aspects such as social isolation and feeling dependent on others. The concerns of children were not expressed by parents and professionals, indicating children might not share their worries with them. However, these worries do occupy their minds and influence their well-being, and Tadić and colleagues conducted semi-structured interviews with children aged 10–15 years in which they also identified “social relationships, participation and acceptance”, “independence and autonomy” and “psychological and emotional well-being” as major themes [21]. Adolescence (age 13–17 years) was marked by a longing for independence and an apparent increase in insecurity, especially towards future life changes. Although elements of social isolation, psychological turmoil, living independently and future employment were also touched upon by professionals and parents, the exact content of the statements of adolescents differs. The finding that the perceptions of adults do not entirely align with the perceptions of children and adolescents has been reported in populations other than those with low vision as well (e.g. in children being hospitalised, attending regular or specialised schools, being part of the general population or having certain behavioural problems [22–24]). The discrepancy between statements of children and adolescents on the one hand, and parents and professionals on the other hand indicate that the real participation needs of children and adolescents are currently not being met. It is therefore necessary for parents and low vision rehabilitation professionals to make the concerns of children and adolescents topics of conversation at home and during the rehabilitative process.

The attitude of parents can have considerable influence on the experiences of a child growing up. To enable healthy development, a child needs a secure environment and sensitive parents, a rich social and physical environment and the opportunity to explore situations independently [25]. However, this supportive environment is not self-evident, because parents’ lives are equally impacted by the disability of the child. The first reaction of parents to the diagnosis of their child, or the intimation that something is wrong, is often compared with bereavement and shock [26, 27]. Consequently, families either adapt to the new situation, which requires a new set of skills and expectations, or find themselves stuck in an emotional state of denial or excessive worrying [4, 28]. These turbulent circumstances in which parents can find themselves might be reflected by the low response rate in the present study. Parents cited several reasons for not participating, including: “I’m too busy to participate”, “I’m already overwhelmed looking after a child with a visual impairment and his/her siblings”, and “I don’t know what my child wants, so I cannot help you with your research”. These answers indicate that MRCs need to be sensitive to the needs and circumstances of parents.

The seeding statements in this study were not limited to only those activities limited by low vision, but were aimed at identifying all activities a child or adolescent would like to participate in, in order to generate a broad overview of activities children and adolescents find important in their lives. The approach used in this study is comparable to the universal application of the ICF-CY, which is also not limited to activities influenced by impairment to identify problems in, for example, participation [16]. Rainey and colleagues evaluated the participation goals of children and adolescents (0–17 years) who registered for rehabilitation services at an MRC and found that most goals related to mobility, prescription of low vision aids and school-related requests [17]. Although the present study shows that these domains are indeed affected by low vision, also other relevant domains were identified. This finding illustrates that currently rehabilitation services do not cover the full scope of issues at hand, potentially limiting visually impaired children and adolescents’ participation.

### Limitations

The results of this study need to be interpreted with some caution. All participating parents were registered at MRCs, which could have biased the information they provided. Nevertheless, it would be nearly impossible to recruit parents of children who never received rehabilitation services, because Dutch healthcare policy stipulates that parents must be informed of the existence of low vision MRCs and their services at the time of diagnosis. Moreover, most children are referred to an MRC to aid development. In addition, most participating children and adolescents attended special education. The impact of low vision on their lives might not be directly generalisable to children attending regular education. It is conceivable that students attending regular education encounter different experiences than students in special needs schools. Although children and adolescents attending regular education were approached to participate in the study, the response rate was very low (5 %). Unfortunately, no information is available on why these students decided not to participate. Future research is needed to confirm whether the conceptual model fits this population as well.

Parents reported a wide variety of causes of low vision for their children. Low vision in children is mostly caused by developmental and genetic disorders, with the most prevalent diagnoses being cerebral visual impairment (CVI), albinism and nystagmus [2]. In our sample, albinism was reported three times and CVI was reported two times by parents, while the remaining 20 children all had different or unknown causes of low vision. However, because extensive data on causes of low vision were lacking, no conclusion can be drawn about the representativeness of this study population with respect to low vision.

To facilitate participation in the study, the methodological approach of the study was specifically tailored to the needs and preferences of the stakeholders. For example, online brainstorm sessions were organised for parents, since it was not logistically feasible to organise face-to-face brainstorm sessions. Several authors have reported using online brainstorm sessions [29]. The tailored methodological approach necessitated the integration of results obtained through four different qualitative methods, which was controlled and systemised by laying down specific *a priori* rules regarding focus group manuals, interview questions and integration methods, thereby limiting information bias.

To develop the conceptual model, the generated statements were clustered within domains. However, statement clustering is liable to subjectivity of the researchers. Some statements could be clustered into multiple domains; for example, the statements “rolling the ball” could be part of the domain “play and sports”, but also of the domain “mobility”. However, the thorough approach regarding statement clustering, which was done by two researchers independently and a third researcher as consultant, resulted in more systematic outcomes.

Last, the conceptual model was not shared with the participants during or after its development, although that is common practice, since this was not in the design of this study. However, a questionnaire will be developed based on this conceptual model, which will be pilot tested to gather some more qualitative information regarding the questionnaire, the underlying conceptual model and performance of the items. Subsequently, psychometric properties of the questionnaire will be investigated using factor analysis and item response theory, thereby testing the structure of the conceptual model.

## Implication and conclusion

The present study provides a solid conceptual model based on which a new questionnaire will be developed, to measure, evaluate and monitor participation needs of children and adolescents with a visual impairment. Our ongoing research focuses on developing and validating this new questionnaire to aid goal setting, rehabilitation referral and, therefore, the successful accomplishment of developmental milestones and life transitions of children and adolescents with a visual impairment, ultimately improving their participation in society and quality of life. Future research should look into the generalisability of the results of this study to children attending regular education and children in other countries.

## Electronic supplementary material

Below is the link to the electronic supplementary material.

Supplementary material 1 (DOCX 73 kb)
