# Supplementary material for: Comprehending the impact of low vision on the lives of children and adolescents: a qualitative approach
Source: Qual Life Res. 2016 Apr 13;25(10):2633–43. doi: 10.1007/s11136-016-1292-8 (PMC5010827; doi:10.1007/s11136-016-1292-8)
Supplement: Supplementary file 1 — Supplementary material 1 (DOCX 73 kb) [file 11136_2016_1292_MOESM1_ESM.docx]

**Table 3** Overview of the generated statements and identified themes per age-band

**a. Statements 0-2 years**

| **Statement^1^** | **Generated by:** | | **Importance of statements by professionals** | **Statements cluster number^2^ by:** | |
| --- | --- | --- | --- | --- | --- |
|  | **Professionals** | **Parents** |  | **Professionals** | **Parents^3^** |
| **Sensory development^4^** | | | | | |
| Preferring toys with sounds | X | X | 7.7 | 11 | 6 |
| Alternating visual attention, for example first looking at mother and then at father | X |  | 8.6 | 13 |  |
| Recognising familiar sounds (for example the door ring, the washing machine, your voice) | X |  | 7.4 | 11 |  |
| Orientation in a room (for example: knows where parent is when he/she is playing) | X | X | 8.1 | 7 | 7 |
| Preferring toys with contrasting colours | X |  | 7.7 | 13 |  |
| Looking to a particular item (for example a toy) | X |  | 8.7 | 13 |  |
| Reacting to light by looking away | X |  | 7.6 | 13 |  |
| Reacting to light by looking into it | X |  | 7.9 | 13 |  |
| Reacting to visual cues (for example hands, toys, carousel) | X |  | 8.3 | 13 |  |
| Reacting to sudden movements (for example being lift up) | X | X | 8.2 | 7 | 6 |
| Preferring shiny toys | X |  | 7.1 | 13 |  |
| Bumping to things often | X |  | 7.5 | 7 |  |
| Exploring items with the mouth | X |  | 7.4 | 11 |  |
| Following a toy or person with the eyes | X |  | 8.6 | 13 |  |
| Understanding of concepts (for example: knows what a table is) | X |  | 7.1 | 12 |  |
| Looking at, for example, pictures for a longer time | X |  | 7.5 | 13 |  |
| Reacting to sounds by looking for it | X | X | 7.5 | 11 | 6 |
| Reacting to (sudden) sounds | X | X | 7.7 | 11 | 6 |
| Exploring the environment by touch | X |  | 8.1 | 11 |  |
| Reacting to light by closing the eyes | X |  | 7.7 | 13 |  |
| **Mobility** | | | | | |
| Sitting | X |  | 8.0 | 1 |  |
| Rolling-over | X |  | 8.1 | 1 |  |
| Jumping | X |  | 5.0 | 1 |  |
| Finding toys in a closet or toy box | X |  | 7.0 | 13 |  |
| Exploring the environment independently | X | X | 8.1 | 7 | 2 |
| Crawling/sliding on buttocks/belly | X | X | 8.2 | 1 | 1 |
| Walking with support | X | X | 7.3 | 1 | 1 |
| Stumbling or falling often | X |  | 7.4 | 1 |  |
| Walking without support | X | X | 7.3 | 1 | 1 |
| Climbing and clambering | X |  | 6.5 | 1 |  |
| Running | X |  | 5.7 | 1 |  |
| Lifting the head | X |  | 7.9 | 1 |  |
| Standing independently | X |  | 7.5 | 1 |  |
| Walking the stairs | X |  | 5.0 | 1 |  |
| Pulling up to stand | X |  | 7.2 | 1 |  |
| **ADL** | | | | | |
| Helping to (un)dress | X |  | 5.7 | 9 |  |
| Eating independently | X | X | 6.2 | 9 | 2 |
| Cleanliness | X |  | 5.5 | 9 |  |
| Drinking independently | X | X | 6.5 | 9 | 2 |
| **Play & sports** | | | | | |
| Completing a simple puzzle | X | X | 6.3 | 12 | 1 |
| Reading a book together with parent | X |  | 7.6 | 7 |  |
| Entertaining his/herself alone | X |  | 7.1 | 10 |  |
| Rolling the ball | X |  | 5.7 | 1 |  |
| Looking at pictures together with parent | X |  | 8.2 | 13 |  |
| Pointing to body parts at his/herself or at a picture (for example nose, eyes) | X |  | 6.8 | 12 |  |
| Playing together with other children of the same age (for example at day-care | X | X | 6.0 | 10 | 5 |
| Manipulating toys (for example press buttons) | X | X | 7.6 | 1 | 1 |
| **Communication** | | | | | |
| Turning head when trying to make contact | X |  | 8.9 | 7 |  |
| Imitating behaviour (for example clapping hands) | X |  | 7.9 | 12 |  |
| Understanding simple gestures (for example pointing or waving) | X |  | 7.8 | 7 |  |
| Imitating sounds | X |  | 6.9 | 11 |  |
| Understanding language (for example when talking to the child) | X |  | 8.5 | 10 |  |
| Talking | X | X | 6.5 | 10 | 3 |
| Recognising facial expressions | X |  | 7.2 | 13 |  |
| Imitating facial expressions | X |  | 7.1 | 11 |  |
| **Social interaction** | | | | | |
| Making contact with other children | X | X | 6.7 | 10 | 4 |
| Recognising faces from familiars (for example parents, family members) | X |  | 8.9 | 13 |  |
| Restless when disappearing from view | X |  | 8.0 | 11 |  |
| **Social-emotional wellbeing** | | | | | |
| Regular day-night cycle | X |  | 8.1 | 10 |  |
| **Parental influence** | | | | | |
| Trusting the child to others (friends, family, babysitter) | X |  | 8.0 | 8 |  |
| **Issues and challenges of parents with a visually impaired child^5^** | | | | | |
| Support from partner | X |  | 8.3 | 8 |  |
| Support from friends or family | X |  | 8.2 | 8 |  |
| Talking about the child with partner | X |  | 8.7 | 8 |  |
| Support from peers | X |  | 6.7 | 8 |  |
| Explaining to others what the visual impairment of the child is | X |  | 7.5 | 8 |  |

^1^ Statements are not an official forward-backward translation
^2^ Cluster numbers: 1=motor skills; 2=ADL; 3=communication; 4=social behaviour; 5=play; 6=sensory perception; 7=orientation; 8=parental support; 9=self-sufficiency; 10=language capacity; 11=sensory development; 12=cognition; 13=visual development ^3^ Statements of parents are clustered by the researchers ^4^ Domains are identical to the domains in the conceptual model
^5^ Issues and challenges of parents are not included in the conceptual model, since they do not directly affect life areas of children and adolescents, but were mentioned by professionals during the concept mapping procedure

**b. Statements 3-6 years**

| **Statement^1^** | **Generated by:** | | **Importance of statements by professionals** | **Statements cluster number^2^ by:** | |
| --- | --- | --- | --- | --- | --- |
|  | **Professionals** | **Parents** |  | **Profes-sionals** | **Parents^3^** |
| **Sensory development^4^** | | | | | |
| Reacting to light by closing the eyes | X |  | 7.9 | 1 |  |
| Reacting to sudden movements (for example being lifted up) | X |  | 6.8 | 3 |  |
| Recognising familiar sounds (for example the door ring, a voice, washing machine) | X |  | 7.8 | 2 |  |
| Recognising colours | X |  | 7.8 | 1 |  |
| Reacting to light by looking away | X |  | 8.0 | 1 |  |
| Bumping to things often | X | X | 7.8 | 2 | 10 |
| Orienting in a room (for example where people are when playing) | X | X | 8.5 | 2 | 9 |
| Exploring items with the mouth | X |  | 7.1 | 2 |  |
| Seeing perspective |  | X |  |  | 10 |
| Following a toy or person with the eyes | X |  | 8.1 | 1 |  |
| Maintaining overview in the classroom | X |  | 7.7 | 1 |  |
| Alternating visual attention to something (for example father and mother) | X |  | 8.1 | 1 |  |
| Reacting to (sudden) sounds | X |  | 7.8 | 1 |  |
| Preferring toys with sounds | X |  | 6.9 | 1 |  |
| Preferring shiny toys | X |  | 6.8 | 1 |  |
| Reacting to light by looking into it | X |  | 7.9 | 1 |  |
| Listening to music | X |  | 5.3 | 3 |  |
| Getting insight in concepts (for example small, big, long, short) | X |  | 8.3 | 2 |  |
| Looking at for example pictures for a longer time | X |  | 7.5 | 1 |  |
| Naming colours | X |  | 7.6 | 1 |  |
| Looking at a particular item (for example a toy) | X |  | 8.7 | 1 |  |
| Reacting to visual cues (for example hands, toys, carousel) | X |  | 8.0 | 1 |  |
| Exploring the environment by touch | X |  | 7.7 | 2 |  |
| Preferring toys with contrasting colours | X | X | 7.2 | 1 | 10 |
| Reacting to sounds by looking for it | X |  | 7.6 | 1 |  |
| **Mobility** | | | | | |
| Cycling | X | X | 6.1 | 6 | 6 |
| Cutting and pasting | X |  | 7.9 | 6 |  |
| Going in the neighbourhood independently | X | X | 6.1 | 4 | 8 |
| Stumbling or falling often | X | X | 7.9 | 6 | 10 |
| Finding toys in a closet or toy box | X |  | 8.1 | 1 |  |
| Finding something in a closet or box independently | X |  | 7.7 | 1 |  |
| Jumping | X | X | 7.1 | 6 | 6 |
| Running | X | X | 7.1 | 6 | 6 |
| Finding food and drinks in a schoolbag | X |  | 6.6 | 1 |  |
| Swimming | X | X | 6.1 | 6 | 6 |
| Finding the way in school | X |  | 7.4 | 2 |  |
| Climbing and clambering | X | X | 7.6 | 6 | 6 |
| Crossing the street independently | X |  | 5.9 | 4 |  |
| Walking without support | X | X | 7.4 | 6 | 6 |
| Walking the stairs | X | X | 7.5 | 6 | 6 |
| Exploring the environment independently | X |  | 8.7 | 4 |  |
| **ADL** | | | | | |
| Drinking independently | X |  | 8.1 | 6 |  |
| (Un)dressing independently | X |  | 7.4 | 6 |  |
| Pouring a drink independently | X |  | 6.0 | 6 |  |
| Tying shoelaces independently | X | X | 5.3 | 6 |  |
| Brushing teeth independently | X |  | 5.6 | 6 |  |
| Finding the coat back independently | X |  | 7.7 | 1 |  |
| Storing items | X |  | 6.3 | 4 |  |
| Wiping buttocks independently | X |  | 5.1 | 4 |  |
| Picking out clothes independently | X |  | 5.2 | 4 |  |
| Eating independently | X |  | 7.8 | 6 |  |
| **Play & sports** | | | | | |
| Playing outside with friends | X | X | 7.3 | 5 | 3 |
| Crafting | X |  | 6.9 | 6 |  |
| Participating in group activities (for example soccer or dancing) | X |  | 7.2 | 5 |  |
| Playing at a friend’s house | X |  | 7.7 | 5 |  |
| Rolling the ball | X |  | 6.3 | 6 |  |
| Manipulating toys (for example push buttons) | X |  | 7.5 | 6 |  |
| Playing a game with rules (for example a board game) | X |  | 6.6 | 5 |  |
| Keeping up with other children while playing | X |  | 7.2 | 3 |  |
| Entertaining him/herself alone | X |  | 7.1 | 5 |  |
| Watching television | X |  | 6.9 | 1 |  |
| Playing imaginary games (for example father-mother games, superhero games) | X |  | 8.0 | 3 |  |
| Playing on playground equipment |  | X |  |  | 3 |
| Completing a puzzle | X |  | 7.6 | 2 |  |
| Playing with other children with a visual impairment | X |  | 6.3 | 5 |  |
| Playing at the playground | X | X | 7.7 | 4 | 3 |
| Using the computer or tablet | X |  | 6.0 | 3 |  |
| Reading books | X |  | 7.6 | 1 |  |
| Participating at other children’s birthday parties | X |  | 7.1 | 5 |  |
| Playing together with other children without a visual impairment | X |  | 7.1 | 5 |  |
| Sporting | X | X | 5.9 | 6 | 6 |
| Using imagination while playing (for example by using voices or making-up stories) | X |  | 7.5 | 3 |  |
| **Communication** | | | | | |
| Asking for help | X |  | 7.5 | 5 |  |
| Sharing experiences (for example at school) | X |  | 6.7 | 5 |  |
| Saying no | X |  | 6.7 | 5 |  |
| Expressing feelings | X |  | 7.1 | 5 |  |
| Recognising facial expressions | X |  | 8.1 | 1 |  |
| Imitating facial expressions | X |  | 7.3 | 3 |  |
| Imitating behaviour (for example clapping hands) | X |  | 7.9 | 2 |  |
| Talking | X |  | 8.1 | 4 |  |
| Turning head when trying to make contact | X |  | 8.1 | 3 |  |
| Asking questions | X |  | 6.9 | 5 |  |
| Stating that he/she wants to join in a group | X |  | 7.0 | 5 |  |
| Imitating sounds | X |  | 5.9 | 4 |  |
| Explaining what he/she can (not) see | X |  | 7.1 | 3 |  |
| Expressing in words properly | X |  | 8.4 | 5 |  |
| **Social interaction** | | | | | |
| Waiting for his/her turn in a group conversation | X |  | 6.1 | 5 |  |
| Recognising faces from familiars (for example parents, family members) | X |  | 8.9 | 1 |  |
| Restless when disappearing from view | X |  | 7.4 | 3 |  |
| Making contact with other children | X |  | 8.4 | 5 |  |
| Inviting a friend to play at home | X |  | 6.9 | 5 |  |
| Empathising with others | X |  | 5.7 | 5 |  |
| Actively participating in a conversation | X |  | 6.6 | 5 |  |
| **Social-emotional wellbeing** | | | | | |
| Realising that he/she is ‘different’ | X |  | 6.4 | 5 |  |
| Regular day-night cycle | X |  | 7.3 | 4 |  |
| Accepting that he/she is ‘different’ | X |  | 6.2 | 5 |  |
| Maintaining enough energy after school for fun activities | X |  | 8.6 | 4 |  |
| **Parental influence** | | | | | |
| Trusting the child to others (family, friends, babysitter) | X | X | 8.1 | 7 | 11 |
| Ability to distance oneself from the child | X |  | 7.7 | 7 |  |
| Protecting the child | X | X | 7.6 | 7 | 11 |
| Taking over difficult tasks from the child | X | X | 7.1 | 7 | 11 |
| **Education/employment** | | | | | |
| Changing from preschool to primary school | X |  | 5.9 | 5 |  |
| Using visual aids | X |  | 6.2 | 3 |  |
| Writing | X |  | 6.1 | 6 |  |
| Participating in physical activity classes | X | X | 7.8 | 6 | 6 |
| Learning how to read braille | X |  | 6.7 | 4 |  |
| **Issues and challenges of parents with a visually impaired child^5^** | | | | | |
| Dividing attention between children | X | X | 7.4 | 7 | 11 |
| Difficulty of the child being ‘different’ | X |  | 8.1 | 7 |  |
| Support from peers | X |  | 6.8 | 7 |  |
| Support from friends or family | X |  | 7.9 | 7 |  |
| Raising the visually impaired child the same way as other children | X | X | 7.4 | 7 | 11 |
| Support from partner | X |  | 7.8 | 7 |  |
| Explaining to others what the visual impairment of the child means | X |  | 8.6 | 7 |  |
| Worrying about the child’s future | X |  | 8.1 | 7 |  |

^1^ Statements are not an official forward-backward translation
^2^ Cluster numbers: 1=visual behaviour; 2=sensory development; 3=play; 4=autonomy; 5=social skills; 6=motor skills; 7=upbringing; 8=mobility outside; 9=orientation; 10=visual perception; 11=ADL
^3^ Statements of parents are clustered by the researchers
^4^ Domains are identical to the domains in the conceptual model
^5^ Issues and challenges of parents are not included in the conceptual model, since they do not directly affect life areas of children and adolescents, but were mentioned by professionals and parents during the concept mapping procedure

**c. Statements 7-12 years**

| **Statement^1^** | **Generated by:** | | | **Importance of statements by professionals** | **Statements cluster number^2^ by:** | | |
| --- | --- | --- | --- | --- | --- | --- | --- |
|  | **Profes-sionals** | **Parents** | **Children** |  | **Profes-sionals** | **Parents^3^** | **Children^3^** |
| **Sensory development^4^** | | | | | | | |
| Reacting to light by closing the eyes | X |  |  | 6.4 | 4 |  |  |
| Reacting to sounds by looking for it | X | X |  | 4.5 | 1 | 15 |  |
| Reacting to loud sounds |  | X | X |  |  | 15 | 15 |
| Reacting to light by looking into it | X |  |  | 4.8 | 4 |  |  |
| Reacting to light by looking away | X | X |  | 5.2 | 4 | 15 |  |
| Getting insight in concepts (for example big, small, long, short) | X |  |  | 8.1 | 4 |  |  |
| Preferring toys with contrasting colours | X |  |  | 5.3 | 4 |  |  |
| Preferring shiny toys | X |  |  | 3.3 | 4 |  |  |
| Bumping to things often | X | X |  | 7.3 | 4 | 1 |  |
| Exploring the environment by touch | X | X |  | 7.7 | 4 | 15 |  |
| Estimating the physical distance to others | X | X |  | 8.2 | 8 | 15 |  |
| Preferring toys with sounds | X |  |  | 3.8 | 3 |  |  |
| Naming colours | X |  |  | 5.3 | 4 |  |  |
| Orienting in a room (for example where people are when playing) | X | X |  | 7.3 | 4 | 15 |  |
| **Mobility** | | | | | | | |
| Going to a club or association independently | X | X |  | 8.3 | 7 | 1 |  |
| Learning a fixed route (for example to school, a friend, or grandparents) | X |  |  | 8.8 | 1 |  |  |
| Becoming ‘traffic-safe’ | X | X | X | 9.1 | 1 | 1 | 1 |
| Finding toys or things in a closet or box | X |  |  | 8.0 | 1 |  |  |
| Walking the stairs | X |  |  | 6.6 | 3 |  |  |
| Finding the way in school independently | X | X |  | 8.7 | 1 | 1 |  |
| Using public transport independently (for example bus, taxi, train) | X |  | X | 6.9 | 1 |  | 1 |
| Stumbling or falling often | X |  |  | 7.8 | 3 |  |  |
| Running | X |  |  | 7.2 | 3 |  | 10 |
| Cycling to a place independently (for example to a friend in the neighbourhood) | X | X | X | 8.4 | 1 | 1 | 1 |
| Finding items in a closet or box independently | X | X |  | 7.8 | 1 | 13 |  |
| Driving a car (in the future) |  |  | X |  |  |  | 1 |
| Driving a scooter (in the future) |  |  | X |  |  |  | 1 |
| Cutting and pasting | X |  |  | 6.3 | 4 |  |  |
| Cycling | X | X | X | 8.6 | 1 | 1 | 1 |
| Walking in the dark |  |  | X |  |  |  | 1 |
| Jumping | X |  | X | 5.9 | 3 |  | 1 |
| Climbing and clambering | X |  |  | 7.3 | 3 |  |  |
| Going to a friend in the neighbourhood independently | X | X | X | 8.1 | 7 | 1 | 1 |
| Finding the way to school | X | X |  | 8.6 | 1 | 1 |  |
| Finding food and drinks in the schoolbag | X |  |  | 6.7 | 1 |  |  |
| Going on a daytrip with family or friends (for example to the zoo or an amusement park) | X |  | X | 7.7 | 5 |  | 18 |
| **ADL** | | | | | | | |
| Recognizing money | X |  |  | 7.4 | 1 |  |  |
| Brushing teeth independently | X | X |  | 7.3 | 2 | 13 |  |
| Doing hairs independently | X | X |  | 7.0 | 2 | 13 |  |
| Pouring a drink independently | X | X |  | 7.3 | 2 | 13 |  |
| Reading the time | X |  |  | 7.0 | 1 |  |  |
| Tying the shoelaces independently | X | X |  | 6.8 | 2 | 13 |  |
| Lubricating a sandwich | X | X |  | 8.3 | 2 | 13 |  |
| Using the mobile phone | X |  | X | 7.8 | 1 |  | 11 |
| Paying independently | X | X |  | 7.4 | 2 | 13 |  |
| Drinking independently | X | X | X | 5.6 | 2 | 13 | 2 |
| (Un)dressing independently | X | X |  | 7.0 | 2 | 13 |  |
| Showering or bathing independently | X | X |  | 8.3 |  | 13 |  |
| Picking out clothes independently | X | X |  | 6.9 | 2 | 13 |  |
| Tidying up the room independently | X | X |  | 7.0 | 2 | 13 |  |
| Using home devices independently (for example TV, DVD-player) | X | X |  | 7.3 | 2 | 13 |  |
| Planning activities | X |  |  | 7.2 | 2 |  |  |
| Handling pocket money | X |  |  | 6.3 | 2 |  |  |
| Going to the toilet independently | X | X |  | 7.7 | 2 | 13 |  |
| Finding the coat back independently | X | X |  | 7.3 | 1 | 13 |  |
| Eating with fork and knife independently | X | X | X | 7.6 | 2 | 13 | 2 |
| **Play & sports** | | | | | | | |
| Swimming | X | X | X | 8.1 | 3 | 10 | 10 |
| Completing a puzzle | X |  |  | 5.5 | 3 |  |  |
| Rolling the ball | X |  | X | 5.9 | 3 |  | 18 |
| Participating in activities with speed (for example cycling fast, roller skating, scooting) | X |  |  | 7.8 | 3 |  |  |
| Drawing or painting |  |  | X |  |  |  | 18 |
| Playing on playground equipment |  |  | X |  |  |  | 18 |
| Playing at a friend’s house | X | X | X | 8.5 | 5 | 11 | 18 |
| Keeping up with other children while playing | X | X | X | 8.3 | 3 | 12 | 14 |
| Listening to music |  |  | X |  |  |  | 18 |
| Making music |  | X | X |  |  | 10 | 18 |
| Manipulating toys (for example push buttons) | X |  |  | 4.8 | 3 |  |  |
| Watching a movie | X |  | X | 6.4 | 4 |  | 18 |
| Crafting | X |  |  | 7.0 | 4 |  |  |
| Gaming |  |  | X |  |  |  | 18 |
| Participating in group activities (for example soccer or dancing) | X | X | X | 8.8 | 9 | 10 | 18 |
| Using the computer or tablet | X | X |  | 8.6 | 1 | 10 |  |
| Sporting | X | X | X | 7.4 | 3 | 10 | 10 |
| Reading books | X | X | X | 8.0 | 4 | 10 | 14 |
| Using social media | X |  | X | 7.8 | 9 |  | 18 |
| Participating at birthday parties | X |  |  | 8.2 | 6 |  |  |
| Playing a game with rules (for example a board game) | X |  | X | 8.1 | 9 |  | 18 |
| Entertaining his/herself alone | X |  | X | 7.3 | 7 |  | 9 |
| Sleeping over | X |  |  | 7.6 | 6 |  |  |
| Using imagination while playing games (for example using voices or making up stories) | X |  |  | 6.6 | 9 |  |  |
| Bungee jumping |  |  | X |  |  |  | 18 |
| Skydiving |  |  | X |  |  |  | 18 |
| Playing together with other children without a visual impairment | X | X | X | 8.8 | 5 | 11 | 18 |
| Playing imaginable games (for example father-mother games, superhero) | X |  |  | 6.7 | 8 |  |  |
| Playing outside with friends | X | X | X | 8.5 | 9 | 11 | 18 |
| Playing with Lego | X |  |  | 5.5 | 3 |  |  |
| **Communication** | | | | | | | |
| Stating he/she wants to join in a group | X |  |  | 8.3 | 6 |  |  |
| Giving an opinion | X |  |  | 7.9 | 6 |  |  |
| Expressing in words properly | X |  |  | 8.0 | 7 |  |  |
| Judging others’ emotions | X | X |  | 8.4 | 8 | 11 |  |
| Sharing experiences | X | X |  | 7.3 | 5 | 17 |  |
| Making jokes | X |  |  | 6.7 | 6 |  |  |
| Saying no | X |  |  | 7.5 | 5 |  |  |
| Participating in a conversation actively | X |  |  | 8.6 | 6 |  |  |
| Recognising facial expressions | X |  |  | 7.4 | 4 |  |  |
| Asking questions | X |  |  | 8.0 | 6 |  |  |
| Indicating what he/she can (not) see | X |  |  | 8.3 | 9 |  |  |
| Asking for help | X |  | X | 8.7 | 6 |  | 17 |
| **Social interaction** | | | | | | | |
| Cooperating with other children | X |  |  | 8.8 | 5 |  |  |
| Making contact with other children | X |  |  | 8.8 | 5 |  |  |
| Meeting new people (children and adults) | X |  |  | 7.5 | 5 |  |  |
| Understanding jokes | X |  |  | 7.3 | 5 |  |  |
| Restless when disappearing from view | X |  |  | 4.8 | 8 |  |  |
| Shopping independently with friends | X | X |  | 6.8 | 7 | 11 |  |
| Inviting a friend to play at home | X |  |  | 8.1 | 6 |  |  |
| Waiting for his/her turn in a group conversation | X |  |  | 7.6 | 5 |  |  |
| Helping other children |  |  | X |  |  |  | 11 |
| Empathising with others | X |  |  | 8.2 | 5 |  |  |
| **Social-emotional wellbeing** | | | | | | | |
| Maintaining enough energy for fun activities | X | X | X | 8.8 | 9 | 12 | 12 |
| Regular day-night cycle | X |  |  | 5.7 | 9 |  |  |
| Dealing with amorousness | X |  |  | 5.8 | 5 |  |  |
| Sharing feelings | X | X |  | 7.9 | 5 | 17 |  |
| Accepting some things are impossible | X | X | X | 8.8 | 8 | 16 | 9 |
| Dealing with visual impairment |  | X | X |  |  | 9 | 9 |
| Making mistakes | X |  |  | 8.1 | 6 |  |  |
| Standing up for him/herself | X |  |  | 8.6 | 5 |  |  |
| Handling bullying from other children | X | X | X | 9.1 | 6 | 11 | 11 |
| Talking about the visual impairment | X |  | X | 8.1 | 9 |  | 9 |
| **Parental influence** | | | | | | | |
| Independence from parents |  | X | X |  |  | 9 | 9 |
| **Education/employment** | | | | | | | |
| Making homework independently | X | X |  | 7.3 | 2 | 14 |  |
| (Un)packing the schoolbag | X |  |  | 6.9 | 1 |  |  |
| Keeping up with classmates | X |  | X | 8.8 | 8 |  | 14 |
| Choosing a high school | X |  |  | 7.1 | 7 |  |  |
| Changing from group or class | X |  |  | 7.4 | 6 |  |  |
| Reading the black board or digital board | X | X | X | 9.1 | 4 | 14 | 14 |
| Participating in physical activity classes | X |  | X | 8.5 | 3 |  | 14 |
| Learning to read braille | X |  |  | 9.1 | 1 |  |  |
| Using visual aids | X |  | X | 8.6 | 9 |  | 14 |
| Changing primary school to secondary school | X |  |  | 8.1 | 7 |  |  |
| Finding information (for example in books or on the internet) | X |  |  | 8.1 | 1 |  |  |
| Becoming a pilot |  |  | X |  |  |  | 1 |
| Maintaining overview in class | X |  |  | 8.2 | 1 |  |  |
| Giving a talk in school | X |  |  | 7.5 | 7 |  |  |
| Writing | X | X | X | 8.3 | 3 | 14 | 14 |

^1^ Statements are not an official forward-backward translation
^2^ Cluster numbers: 1=mobility; 2=self-care; 3=motor skills; 4=visual functioning; 5=sexual development; 6=social skills; 7=cope with change; 8=play; 9=acceptance; 10=sports and hobbies; 11=social interaction; 12=energy; 13=ADL; 14=school; 15=sensory perception 16=social-emotional; 17-communication; 18=leisure time;
^3^ Statements of parents and children are clustered by the researchers
^4^ Domains are identical to the domains in the conceptual model

**d. Statements 13-17 years**

| **Statement^1^** | **Generated by:** | | | **Importance of statements by professionals** | **Statements cluster number^2^ by:** | | |
| --- | --- | --- | --- | --- | --- | --- | --- |
|  | **Profes-sionals** | **Parents** | **Adole-scents** |  | **Profes-sionals** | **Parents^3^** | **Adole-scents^3^** |
| **Sensory development^4^** | | | | | | | |
| Bumping to things often |  | X |  |  |  | 11 |  |
| Estimating the physical distance to others | X |  |  | 8.4 | 7 |  |  |
| Orienting in a room (for example knowing where people are) | X |  | X | 8.4 | 3 |  | 11 |
| **Mobility** | | | | | | | |
| Going to a club or association independently | X | X | X | 8.8 | 7 | 11 | 11 |
| Learning a fixed route | X |  |  | 8.8 | 3 |  |  |
| Participating in traffic independently at night (when it is dark) | X | X | X | 7.8 | 3 | 11 | 11 |
| Participating in traffic during day-time | X | X | X | 8.8 | 3 | 11 | 11 |
| Walking the stairs | X |  |  | 5.2 | 4 |  |  |
| Crossing the street independently | X | X | X | 8.7 | 3 | 11 | 11 |
| Running | X |  |  | 5.0 | 4 |  |  |
| Moving out |  |  | X |  |  |  | 11 |
| Finding the way in school independently | X |  | X | 9.1 | 3 |  | 11 |
| Going on holiday with friends (for example for a weekend) | X |  | X | 7.0 | 7 |  | 11 |
| Cycling to a place independently (for example to a friend in the neighbourhood) | X | X | X | 8.2 | 3 | 11 | 11 |
| Finding the way to school independently | X |  | X | 8.7 | 3 |  | 11 |
| Finding items in for example a closet | X |  |  | 8.3 | 1 |  |  |
| Finding items in the closet or on the desk independently | X |  |  | 8.7 | 1 |  |  |
| Driving motor |  |  | X |  |  |  | 11 |
| Cycling | X | X | X | 8.3 | 3 | 11 | 11 |
| Walking in the dark | X |  | X | 8.0 | 3 |  | 11 |
| Jumping | X |  |  | 4.0 | 4 |  |  |
| Going to a friend in the neighbourhood independently | X | X | X | 8.3 | 3 | 11 | 6 |
| Going into the neighbourhood independently | X | X | X | 8.8 | 3 | 11 | 11 |
| Going on a daytrip with family or friends (for example to an amusement park or festival) | X |  | X | 8.3 | 6 |  | 9 |
| **ADL** | | | | | | | |
| Recognising money | X |  |  | 7.6 | 1 |  |  |
| Doing hair independently | X |  |  | 7.6 | 2 |  |  |
| Pouring a drink independently | X |  |  | 7.6 | 1 |  |  |
| Lubricating a sandwich independently | X |  |  | 7.0 | 2 |  |  |
| Taking medication independently | X |  |  | 7.5 | 2 |  |  |
| Living independently | X | X | X | 6.6 | 2 | 15 | 11 |
| Make the bed | X |  |  | 6.7 | 2 |  |  |
| Managing bank account | X |  |  | 7.3 | 2 |  |  |
| Processing the post | X |  |  | 7.3 | 2 |  |  |
| Shopping groceries (for example drinks, food or candy) | X |  | X | 8.5 | 1 |  | 2 |
| Doing the dishes or packing the dishwasher | X |  | X | 7.3 | 2 |  | 2 |
| Doing make-up independently | X |  |  | 7.8 | 1 |  |  |
| Handling the menstruation | X |  |  | 8.2 | 2 |  |  |
| Cooking independently | X |  | X | 7.5 | 2 |  | 2 |
| Reading signs |  |  | X |  |  |  | 11 |
| Doing the laundry |  |  | X |  |  |  | 2 |
| Using the mobile phone | X |  | X | 8.6 | 1 |  | 13 |
| Paying independently | X |  |  | 8.1 | 2 |  |  |
| Showering or bathing independently | X |  |  | 7.8 | 2 |  |  |
| Picking out clothes independently | X |  |  | 7.8 | 1 |  |  |
| Tidying up the room independently | X |  | X | 7.4 | 2 |  | 2 |
| Using home devices (for example TV, DVD player, washing machine) | X |  | X | 8.7 | 2 |  | 2 |
| Planning activities | X |  |  | 8.8 | 1 |  |  |
| Handling pocket money | X |  |  | 7.9 | 2 |  |  |
| Finding the coat back independently | X |  |  | 7.7 | 1 |  |  |
| Finding the locker back independently | X |  |  | 8.3 | 1 |  |  |
| Eating with fork and knife independently | X |  |  | 7.1 | 2 |  |  |
| **Play & sports** | | | | | | | |
| Completing a puzzle | X |  |  | 3.3 | 4 |  |  |
| Swimming | X |  | X | 5.7 | 3 |  | 12 |
| Participating in activities with speed (for example cycling fast, roller skating or scooting) | X |  | X | 6.7 | 4 |  | 11 |
| Drawing | X |  |  | 4.3 | 4 |  |  |
| Keeping up with others while playing/doing sports | X | X | X | 7.8 | 5 |  | 12 |
| Playing at a friend’s house | X | X | X | 7.6 | 7 | 6 | 6 |
| Playing outside |  |  | X |  |  |  | 9 |
| Listening to music |  |  | X |  |  |  | 9 |
| Making music |  |  | X |  |  |  | 9 |
| Doing activities in leisure time | X |  |  | 8.4 | 2 |  |  |
| Woodworking |  |  | X |  |  |  | 9 |
| Lighting fireworks |  |  | X |  |  |  | 9 |
| Fishing |  |  | X |  |  |  | 9 |
| Acting |  |  | X |  |  |  | 9 |
| Watching a movie | X |  | X | 6.2 | 3 |  | 9 |
| Crafting | X |  |  | 3.8 | 3 |  |  |
| Gaming |  |  | X |  |  |  | 9 |
| Participating in group activities (for example soccer or dancing) | X | X | X | 8.3 | 7 | 6 | 12 |
| Using the computer or tablet | X |  |  | 9.1 | 3 |  |  |
| Doing sports | X | X | X | 8.0 | 3 | 12 | 12 |
| Reading books | X | X | X | 8.0 | 3 | 10 | 10 |
| Using social media | X |  | X | 9.0 | 3 |  | 6 |
| Participating at (birthday) parties | X |  |  | 8.1 | 8 |  |  |
| Playing a game with rules | X |  | X | 6.7 | 6 |  | 6 |
| Playing or doing activities with other children without a visual impairment | X | X | X | 7.8 | 8 | 6 | 6 |
| Sleeping over | X |  |  | 6.9 | 7 |  |  |
| Entertaining him/herself alone | X |  |  | 7.0 | 7 |  |  |
| **Communication** | | | | | | | |
| Giving an opinion | X |  |  | 8.6 | 8 |  |  |
| Expressing in words properly | X |  |  | 8.1 | 8 |  |  |
| Judging others’ emotions | X |  |  | 8.7 | 5 |  |  |
| Sharing experiences | X |  |  | 7.7 | 6 |  |  |
| Stating he/she wants to join a group | X | X |  | 8.6 | 6 | 6 |  |
| Talking about driving a car | X |  | X | 7.2 | 5 |  | 11 |
| Indicating what he/she can (not) see | X | X | X | 8.5 | 5 | 5 | 14 |
| Recognising facial expressions | X |  |  | 8.1 | 5 |  |  |
| Talking with friends |  |  | X |  |  |  | 6 |
| Talking with classmates |  |  | X |  |  |  | 13 |
| Talking with teachers |  |  | X |  |  |  | 13 |
| Making eye contact with others |  |  | X |  |  |  | 13 |
| Making jokes | X |  |  | 7.5 | 8 |  |  |
| Saying no | X |  | X | 8.8 | 8 |  | 5 |
| Participating in a conversation actively (for example during dinner) | X |  | X | 8.6 | 8 |  | 6 |
| Asking questions | X |  |  | 8.2 | 6 |  |  |
| Asking for help | X |  | X | 8.9 | 7 |  | 13 |
| **Social interaction** | | | | | | | |
| Cooperating with others | X |  |  | 8.4 | 6 |  |  |
| Making contact with other children | X |  | X | 8.9 | 6 |  | 6 |
| Meeting new people (adolescents and adults) | X | X |  | 8.4 | 6 | 6 |  |
| Understanding jokes | X |  |  | 7.6 | 6 |  |  |
| Shopping independently with friends | X |  | X | 8.6 | 7 |  | 6 |
| Receiving help from peers |  |  | X |  |  |  | 6 |
| Going out for dinner |  |  | X |  |  |  | 9 |
| Contacting peers |  |  | X |  |  |  | 6 |
| Dating | X |  | X | 8.1 | 8 |  | 6 |
| Having a romance with a partner | X |  | X | 7.3 | 8 |  | 6 |
| Going a night out with friends (for example to the cinema, a theatre or bar) | X | X | X | 8.3 | 7 | 15 | 9 |
| Flirting | X | X | X | 7.5 | 6 | 15 | 6 |
| Inviting a friend to come at home | X | X | X | 7.7 | 6 | 6 | 6 |
| Waiting for his/her turn in a group conversation | X |  |  | 7.9 | 6 |  |  |
| Empathising with others | X |  |  | 8.0 | 6 |  |  |
| **Social-emotional wellbeing** | | | | | | | |
| Maintaining enough energy after school for fun activities | X | X | X | 8.6 | 5 | 16 | 8 |
| Wanting to be ‘normal’ |  | X | X |  |  | 5 | 5 |
| Knowing what is right |  |  | X |  |  |  | 5 |
| Being afraid to do things independently |  |  | X |  |  |  | 5 |
| Dealing with amorousness | X |  | X | 8.1 | 8 |  | 6 |
| Sharing feelings | X |  |  | 7.8 | 6 |  |  |
| Accepting some things are impossible | X | X | X | 9.2 | 5 | 5 | 5 |
| Making mistakes | X |  |  | 7.7 | 6 |  |  |
| Standing up for him/herself | X |  | X | 8.8 | 6 |  | 5 |
| Handling bullying from other children | X |  | X | 8.8 | 6 |  | 6 |
| Talking about the visual impairment (for example during a presentation in class) | X | X | X | 8.4 | 5 | 5 | 13 |
| Being insecure |  | X | X |  |  | 15 | 8 |
| **Parental influence** | | | | | | | |
| Overprotecting parents |  |  | X |  |  |  | 14 |
| Being dependent on others | X | X | X | 8.3 | 5 | 5 | 5 |
| Worrying parents |  |  | X |  |  |  | 14 |
| **Education/employment** | | | | | | | |
| Making homework independently | X |  | X | 8.5 | 2 |  | 10 |
| Keeping up with classmates | X |  | X | 8.7 | 5 |  | 10 |
| Choosing an appropriate study | X |  | X | 8.9 | 2 |  | 10 |
| Changing class (for example from first to second class) | X |  | X | 7.6 | 7 |  | 10 |
| Reading the black board or digital board | X |  | X | 8.6 | 3 |  | 10 |
| Learning to read braille | X |  |  | 6.9 | 3 |  |  |
| Using visual aids | X | X | X | 9.2 | 1 | 10 | 10 |
| Finding information (for example on internet or in books) | X |  |  | 9.1 | 1 |  |  |
| Maintaining overview in the class room | X |  |  | 8.1 | 3 |  |  |
| Participating in physical activity classes | X |  |  | 8.2 | 3 |  |  |
| Writing |  |  | X |  |  |  | 10 |
| Participating in excursions | X |  |  | 8.0 | 7 |  |  |
| Choosing an appropriate part-time or holiday job | X |  | X | 7.7 | 7 |  | 10 |
| Giving a presentation | X |  |  | 6.9 | 7 |  |  |
| Achieving a diploma | X |  |  | 8.7 | 7 |  |  |
| Receiving assistance at work |  |  | X |  |  |  | 11 |
| Learning new tasks |  |  | X |  |  |  | 10 |

^1^ Statements are not an official forward-backward translation
^2^ Cluster numbers: 1=autonomy; 2=ADL; 3=traffic participation; 4=motor skills; 5=acceptance; 6=social interaction; 7=cope with change; 8=social-emotional wellbeing; 9=leisure time activities; 10=school/work; 11=mobility; 12=sports; 13=communication; 14=overprotection; 15=social emotional development; 16=energy
^3^ Statements of parents and children are clustered by the researchers
^4^ Domains are identical to the domains in the conceptual model
